# A Case of Enteritis

**Published:** 1884-10

**Authors:** Alvah C. Van Syckle

**Affiliations:** Hackettstown, New Jersey


### II.—A CASE OF ENTERITIS.

BY ALVAH C. VAN SYCKLE, M.D.

In order to give a careful history of this case, I should state that there has been prevailing in this locality an infectious disease resembling distemper.

It comes under a nomenclature of the scarlet fever type in childhood. Most of the horses begin with malaise, followed in 48 hours with a cough,

*From Wochenschrift für Thierheilkunde. *No.* 24, 1884.

enlargement of the parotid and submaxillary glands, slight muco-purulent pus from the nostrils, loss of appetite, accompanied with high fever. In some cases the discharge at the nostril does not manifest itself, but seems to be transmitted to the thoracic or abdominal organs. This latter symptom manifested itself in the case which I am about to relate. Last Saturday, July 5th, I was called to see a valuable 5-year-old trotter owned at the Speedlawn Stock Farm of Asa W. Trainner. She had been and was scouring fearfully, temperature not taken, pulse 75, had been sick about one week, no appetite but great thirst, no running at the nose, slight enlargement of the glands. Ordered quinia and astringent clysters; as another gentleman was attending the case, I gave no further directions. On July 7th, Drs. J. Stock, Nelden and myself were called to see her. We gave as our diagnosis enteritis complicating the peritoneum. The pulse at this time, 4 P. M., was 84, eyelids congested, mucous membrane of nostrils tense and red, bloating perceptible, limbs cold, flanks heaving laboriously, no voiding of urine, scouring ceased. Dr. Nelden ordered:

℞
Tinct. Belladon. rad.
Tinct. Aconit. āā

♏. To be given every two hours for the fever.

As an anodyne:

℞
Pulv. Opii.
Pulv. Camph.
Quinia sulph.

♏.

On Tuesday morning, 1 o'clock, she died.

*Necropsy.*—The mare was bloated, the skin tense, but on section the adipose tissue was quite prominent. Thoracic cavity.—The heart was healthy as far as examined.. The aorta was black with blotches. Lungs,—showed slight congestion; on transverse section the lung tissue was normal. Kidneys,—both organs were healthy, but in the tubes muco-pus was found similar to that which passes from the nostrils. Stomach,—contained about four quarts of liquid. Small intestines filled with gases, also inflammation was noticed. Large intestines were in a state of inflammation. Peritoneum.—Evidences were present showing that peritonitis had set in. Liver.—Small ecchymotic spots were observed on the liver.

*Summary.*—Here we have a disease fatal, yet so insidious is it that the best veterinarian is apt to be misled as to its seriousness. It teaches us that the treatment must be (1) tonic, (2) sedative. The proper medicine to use is Opii to control the peritoneal fluid, and then Quinia sulphate as a febrifuge and tonic. Aconite is a good arterial sedative, and should be given only as far as the results of reducing the temperature and no further. (3) External fomentations, or better, counter-irritation should be employed as soon as possible; these with good hygienic surroundings is all that we can do. The mortality of these cases seems to be two out of five.

Hackettstown, New Jersey.

